# Gestational weight gain and increased risk of cesarean delivery across body mass index categories

**DOI:** 10.1016/j.xagr.2025.100445

**Published:** 2025-01-17

**Authors:** Pearl A. McElfish, Britni L. Ayers, Nicola L. Hawley, Aaron Caldwell, Austin Porter, Michael D. Macechko, Donya Watson, Jennifer A. Callaghan-Koru, James P. Selig, Jennifer A. Andersen, Nirvana Manning, Lanita White, Enrique Gomez-Pomar, Clare C. Brown

**Affiliations:** 1 College of Medicine, University of Arkansas for Medical Sciences Northwest, Springdale, AR (McElfish, Callaghan-Koru, and Andersen); 2 College of Nursing, University of Arkansas for Medical Sciences Northwest, Fayetteville, AR (Ayers); 3 Department of Chronic Disease Epidemiology, Yale School of Public Health, New Haven, CT (Hawley); 4 Fay W. Boozman College of Public Health, University of Arkansas for Medical Sciences Northwest, Springdale, AR (Caldwell, Callaghan-Koru, and Selig); 5 Fay W. Boozman College of Public Health, University of Arkansas for Medical Sciences, Little Rock, AR (Porter and Brown); 6 Arkansas Department of Health, Little Rock, AR (Porter); 7 College of Medicine, University of Arkansas for Medical Sciences Northwest, Fayetteville, AR (Macechko); 8 College of Medicine, University of Arkansas for Medical Sciences, Little Rock, AR (Watson, Manning, and Gomez-Pomar); 9 South Arkansas Regional Hospital, El Dorado, AR (Watson); 10 Community Health Centers of Arkansas, North Little Rock, AR (White); 11 Neonatology, St. Bernards Regional Medical Center, Jonesboro, AR (Gomez-Pomar).

**Keywords:** body mass index, cesarean delivery, excessive gestational weight gain, gestational weight gain, maternal morbidity

## Abstract

**BACKGROUND:**

Unnecessary cesarean delivery can have negative implications for both mothers and infants. In the United States, the proportion of women undergoing cesarean delivery exceeds the acceptable World Health Organization proportion. Reducing cesarean deliveries is a national goal of Centers for Disease Control and Prevention Healthy People 2030, the American College of Obstetricians and Gynecologists, and the Alliance for Innovation on Maternal Health.

**OBJECTIVE:**

This study aimed to examine if excessive gestational weight gain is associated with increased risk of cesarean delivery across multiple body mass index categories.

**STUDY DESIGN:**

Analysis was conducted using vital records data from the National Center for Health Statistics birth records. Only low-risk births were included (singleton, term-gestation [≥37 weeks], cephalic presentation, and first birth to exclude women who had a prior cesarean delivery). We used the rate of gestational weight gain (lb/wk) measured as both a categorical and continuous variable. These results were confirmed by a sensitivity analysis using total gestational weight gain (lb).

**RESULTS:**

Regardless of prepregnancy body mass index category, women with excessive gestational weight gain had a higher risk of cesarean delivery. Among women with a healthy prepregnancy body mass index, the risk of cesarean delivery decreased with appropriate weight gain, suggesting a potential protective effect of moderate weight gain for individuals with a healthy prepregnancy body mass index. However, weight gain beyond the appropriate level increased the risk of cesarean delivery. For women with overweight or obese prepregnancy body mass index, any increase in gestational weight gain was associated with a higher cesarean delivery risk.

**CONCLUSION:**

This study found a strong association between an excessive rate of gestational weight gain and the risk of cesarean delivery, regardless of prepregnancy body mass index, suggesting the need for continued efforts to reduce excessive gestational weight gain across populations.

**AJOG Global Reports at a Glance**

**Why was this study conducted?**

In the United States, the proportion of women who have a cesarean delivery is significantly higher than recommended.

**Key findings**

Women with excessive gestational weight gain had a higher risk of cesarean delivery, regardless of prepregnancy body mass index category.

**What does this add to what is known?**

This study analyzed multiple prepregnancy body mass index categories (not only overweight and obesity). Furthermore, it examined cesarean delivery outcomes using the rate of gestational weight gain, including its analysis as a continuous variable. These results were confirmed in our sensitivity analysis using total gestational weight gain.

## Introduction

The United States has higher maternal morbidity and mortality than other high-income nations,^1^^,^^2^ and Arkansas has some of the highest rates of maternal morbidity and mortality in the United States.^3^^,^^4^ One of the primary drivers of severe maternal morbidity is cesarean delivery.^5^, ^6^, ^7^ In the Unites States, the percentage of women having a cesarean delivery has significantly increased from 7% in 1990 to 32% in 2023, exceeding the World Health Organization's acceptable percentage of 10% to 15%.^8^, ^9^, ^10^ In 2023, approximately 34% of all births in Arkansas were cesarean deliveries, which is higher than the national average.^11^

Although cesarean delivery can reduce both maternal and neonatal morbidity and mortality when conducted for appropriate medical indications, it can have long-term negative consequences for the mother and the infant when performed unnecessarily.^6^ Cesarean delivery is linked to increased early maternal complications such as hemorrhage resulting in transfusion, major infection, shock, uterine rupture, placenta previa, hysterectomy, and complications in subsequent pregnancies.^7^ For infants, epidemiologic studies have documented associations of cesarean delivery with increases in the frequency of noncommunicable diseases such as asthma, food allergies, and obesity.^7^ In addition, infants born via cesarean delivery are not exposed to the variety of microorganisms associated with vaginal birth, which are beneficial to the development of the immune system.^12^ Reducing cesarean delivery rates is a national goal of the Centers for Disease Control and Prevention Healthy People 2030, the American College of Obstetricians and Gynecologists (ACOG), and the Alliance for Innovation on Maternal Health.^13^^,^^14^

The National Academy of Medicine (NAM) and the World Health Organization recommend gestational weight gain (GWG) guidelines tailored to prepregnancy body mass index (BMI). These recommendations aim to mitigate the risks associated with excessive or insufficient GWG to promote healthy outcomes for both the mother and infant.^15^^,^^16^ Some studies have documented an association between excessive gestational weight gain (EGWG) and cesarean delivery; however, these studies had small sample sizes, and most were conducted within only 1 racial/ethnic group.^17^, ^18^, ^19^ Furthermore, prior studies have not examined whether the risk of cesarean delivery associated with EGWG differs across the full range of BMI categories, including the multiple classes of obesity. Given the increase in the percentage of women with EGWG and increases in prepregnancy obesity rates,^20^^,^^21^ understanding the interactions between EGWG and prepregnancy BMI may provide important information for reducing the high proportion of cesarean delivery in the United States. Therefore, understanding the factors associated with cesarean delivery, particularly among low-risk pregnancies, is critical for the health of mothers and infants.

Many nonclinical factors associated with cesarean delivery (maternal age, race, socioeconomic status, and insurance coverage) and clinical factors associated with cesarean delivery (prepregnancy BMI and prepregnancy health condition) are challenging to modify.^20^^,^^22^, ^23^, ^24^ However, GWG may be modifiable. Therefore, the purpose of this research was to examine whether EGWG was associated with increased risk of cesarean delivery across multiple BMI categories of women in Arkansas, which has the second highest prevalence of obesity among women in the United States,^25^ with ∼60% to 68% of women in Arkansas having overweight or obesity when they become pregnant.^26^, ^27^, ^28^, ^29^

## Methods

### Data and population

Vital records data from the National Center for Health Statistics birth records were used. The study population consisted of singleton live births of mothers who were residents of Arkansas between January 1, 2014, and December 31, 2022. Given our goal to examine the effects of EGWG on cesarean delivery in low-risk pregnancies, only live births that met the following criteria were included: singleton, reached term (gestational age ≥37 weeks), cephalic presentation at birth, and first birth in the total birth order to exclude women who had a prior cesarean delivery.

### Variable definitions

The primary outcome of interest was the method of delivery (cesarean vs vaginal delivery). The main risk factor of interest was EGWG, defined by the rate of GWG in the second and third trimesters. Although many epidemiologic studies have focused on total weight gain, evaluating the rate of weight gain (which is also represented in the NAM guidelines^15^) provides a more nuanced assessment, as it allows us to consider whether a woman's weight gain aligns with expected patterns, accounting for natural variations in gestational length.^30^ For example, a woman with a delivery at 40 weeks may naturally be expected to gain more weight than a woman with a delivery at 37 weeks. In addition, we completed a sensitivity analysis using total weight gain, which is included in the Supplemental Appendix.

The rate of weight gain was categorized as insufficient, healthy, or excessive according to whether the GWG per week was below, within, or greater than the current recommended ranges (underweight: 1.0–1.3 lb/wk; healthy: 0.8–1.0; overweight: 0.5–0.7; and obesity class I–III: 0.4–0.6 lb/wk) based on prepregnancy BMI. The rate of GWG (GWG per week) for the second and third trimesters was calculated by the method previously used by Wu et al^31^ (2024), wherein 2.75 lb is the midpoint of the suggested GWG range (1.1–4.4 lb) for the first trimester^15^ and 12 completed weeks is the length of the first trimester.

$$Rate\;of\;GWG=\frac{Total\;GWG-2.75\;lb}{Gestational\;age-12\;weeks}$$

We adjusted for demographic variables in the analysis. Payer was defined as the payer for the delivery and included Medicaid, private insurance, other (eg, Indian Health Service or TRICARE), and self-pay. Maternal race/ethnicity was coded as Hispanic (regardless of race); non-Hispanic American Indian or Alaska Native; Asian; White; Black; Native Hawaiian or Pacific Islander; or multiracial. The county of residence of the birth mother was coded as being “metro” (ie, urban) or “non-metro” (ie, rural) using the 2023 Rural-Urban Continuum Codes from the United States Department of Agriculture. Birth year (ie, 2014–2022) was included as a fixed effect. Additional covariates included other maternal risk factors. Specifically, we included maternal age (continuous), gestational age (37–44 weeks), prepregnancy BMI category (underweight, healthy, overweight, obesity I, obesity II, or obesity III), diabetes (prepregnancy and gestational), hypertension (prepregnancy and gestational), and labor induction.

### Statistical analyses

We used a generalized linear model (GLM) with a binomial distribution and a logit link to model cesarean delivery outcomes. We estimated marginal predicted probabilities and calculated average absolute risk differences (RD). The GLM included all covariates, the main effect of EGWG, and an interaction between prepregnancy BMI and EGWG. The interaction was included because of the potential modification of EGWG effects by prepregnancy BMI (ie, a multiplicative effect of GWG with BMI). We fit generalized additive models to analyze GWG as a continuous variable, using the “mgcv” R package with thin-plate regression splines with a smooth-factor interaction between BMI category and GWG.^32^ From this model, the smooth terms (splines) can be interpreted using the effective degrees of freedom (edf) to measure the complexity of the smooth term in the model. A higher edf implies a more complex, “wiggly” curve capable of capturing finer details in the relationship between the predictor (rate of GWG) and the response variable (cesarean delivery).^32^ If the edf for a smooth term is substantially different across levels of the interacting factor (ie, BMI categories), it suggests that the relationship between the predictor (rate of GWG) and response (cesarean delivery) is markedly different for those factor levels. Predicted probabilities, absolute RDs, and the partial derivative of the slopes were calculated and visualized using the “marginaleffects” R package.^33^ Additional details of our analysis are included in the Supplemental Appendix, including the regression coefficients and other model information. Statistical significance was set at *P*<.05, and 95% confidence intervals were reported. All analyses were conducted using R version 4.3.2 (R Foundation for Statistical Computing, Vienna, Austria).^34^

## Results

A total of 87,795 births met our study criteria, and 4993 births had some missing data. Of these, 3247 were missing some form of weight-related data (ie, prepregnancy weight or weight gained during pregnancy). We conducted a complete case analysis of 82,802 singleton live births in Arkansas. This included first births in the total birth order to women who reached term (gestational age ≥37 weeks), whose infants had a cephalic presentation at birth (ie, not breech), and who did not have missing data on the outcomes or covariates. Table 1 presents the characteristics of the study population. In our sample, approximately 25.8% of births were by cesarean delivery, and 63.3% of women had an excessive rate of GWG (details provided in the Supplemental Appendix).

**Table 1:** Characteristics of the study population of 82,802 singleton births

Characteristic	Insufficient N=19,296	Healthy N=11,123	Excessive N=52,383	*P* value^a^
Delivery method				<.001
Spontaneous	72%	72%	65%	
Forceps	1.1%	1.1%	1.0%	
Vacuum	5.4%	5.5%	5.7%	
Cesarean	21%	21%	28%	
Gestational age at birth				<.001
Mean (SD)	39.56 (1.77)	39.60 (1.77)	39.34 (1.55)	
Prepregnancy BMI				<.001
Underweight	5.9%	8.3%	2.5%	
Healthy	47%	51%	41%	
Overweight	17%	18%	28%	
Obesity I	12%	11%	16%	
Obesity II	8.9%	6.1%	7.2%	
Obesity III	9.1%	5.1%	4.7%	
Mother's age, y				<.001
<20	22%	19%	19%	
20–24	37%	36%	39%	
25–29	25%	27%	27%	
30–34	12%	14%	12%	
35–39	3.4%	3.7%	3.1%	
≥40	0.8%	0.6%	0.4%	
Prepregnancy diabetes	0.6%	0.4%	0.6%	.2
Prepregnancy hypertension	1.6%	1.1%	1.7%	<.001
Gestational diabetes	5.2%	4.4%	3.6%	<.001
Gestational hypertension	5.4%	5.8%	10%	<.001
Induction of labor	38%	39%	46%	<.001

*BMI*, body mass index.

a Pearson chi-squared test; Kruskal–Wallis rank sum test.

**Figure 1:**
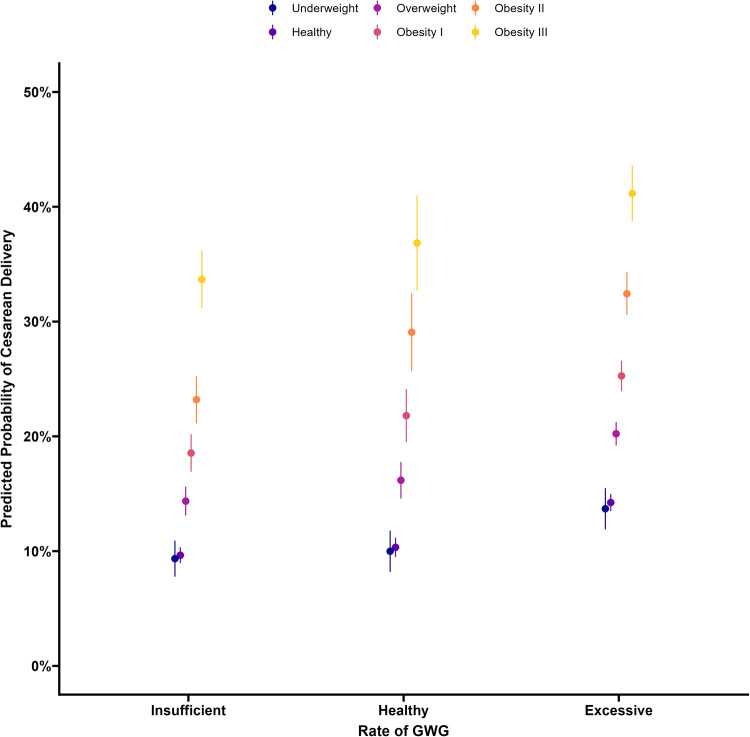
Predicted population average probability of cesarean delivery with 95% CIs *CI*, confidence interval; *GWG*, gestational weight gain.

Figure 1 displays the average predicted probability of cesarean delivery by BMI category and rate of GWG categorization averaged over covariates. Inspection of the regression coefficients indicated no interaction between GWG and BMI (all *P*>.05), indicating no multiplicative effect of rate of GWG categorization with BMI.

McElfish. Gestational weight gain and increased risk of cesarean delivery. Am J Obstet Gynecol Glob Rep 2025.

Higher BMI was associated with a higher risk of cesarean delivery (ie, main effect of prepregnancy BMI) (Table 2). There was no significant difference in the average risk of cesarean delivery between underweight and healthy-weight birthing mothers (*P*=.45). However, each successive increase in prepregnancy BMI category was associated with a 5% to 7% increase in the risk of cesarean delivery (Table 2). For example, birthing mothers who were overweight had a 7.33% increase (*P*<.001) in the risk of cesarean delivery compared with mothers with a healthy prepregnancy body weight.

**Table 2:** Contrasts of the risk difference between gestational weight gain categories

Variable	Contrast	RD	z	*P* value	95% CI
BMI					
	Healthy–Underweight	0.61%	0.75	.451	−0.98%–2.20%
	Overweight–Healthy	7.33%	19.58	<.001	6.60%–8.06%
	Obesity I–Overweight	5.98%	11.43	<.001	4.95%–7.00%
	Obesity II–Obesity I	7.51%	10.11	<.001	6.05%–8.97%
	Obesity III–Obesity II	9.61%	9.94	<.001	7.71%–11.50%
Weight gain rate					
	Excessive–Healthy	4.78%	10.68	<.001	3.90%–5.66%
	Excessive –Insufficient	7.09%	20.14	<.001	6.40%–7.78%
	Healthy –Insufficient	2.31%	4.62	<.001	1.33%–3.28%

*BMI*, body mass index; *CI*, confidence interval; *RD*, risk difference.

Women with an excessive rate of GWG had a significantly higher risk of cesarean delivery than those with a healthy GWG rate (4.78%; *P*<.001) (Table 2) or an insufficient GWG rate (7.09%; *P*<.001). Mothers with a healthy GWG rate (2.31%; *P*<.001) had a slightly elevated risk compared with mothers with an insufficient GWG rate.

Our sensitivity analysis (Supplemental Appendix) confirmed our main findings: women with total EGWG (lb) had a significantly higher risk of cesarean delivery than those with a healthy total GWG (6.08%; *P*<.001) or insufficient total GWG (7.85%; *P*<.001). Similar to our findings related to the rate of GWG, mothers with a healthy total GWG (1.77%; *P*<.001) had a slightly higher risk of cesarean delivery than mothers with insufficient GWG.

### Continuous analysis of rate of gestational weight gain and visualization

Generalized additive models with regression splines were used to analyze the nonlinear relationship between the GWG rate as a continuous variable and the risk of cesarean delivery. Inspection of the smooth terms indicated a high degree of complexity for the relationship between BMI and GWG rate (edf=3.67; *P*<.001). Both the healthy (edf=6.09; *P*<.001) and underweight (edf=3.57; *P*=.14) groups showed a high degree of complexity beyond the overall trend; however, the interaction between BMI and GWG rate was nonsignificant for the underweight group. All other smooth factor interactions were compatible with the overall smooth trend for BMI and GWG rate (all edf=1; all *P*>.30) (Supplemental Appendix).

**Figure 2:**
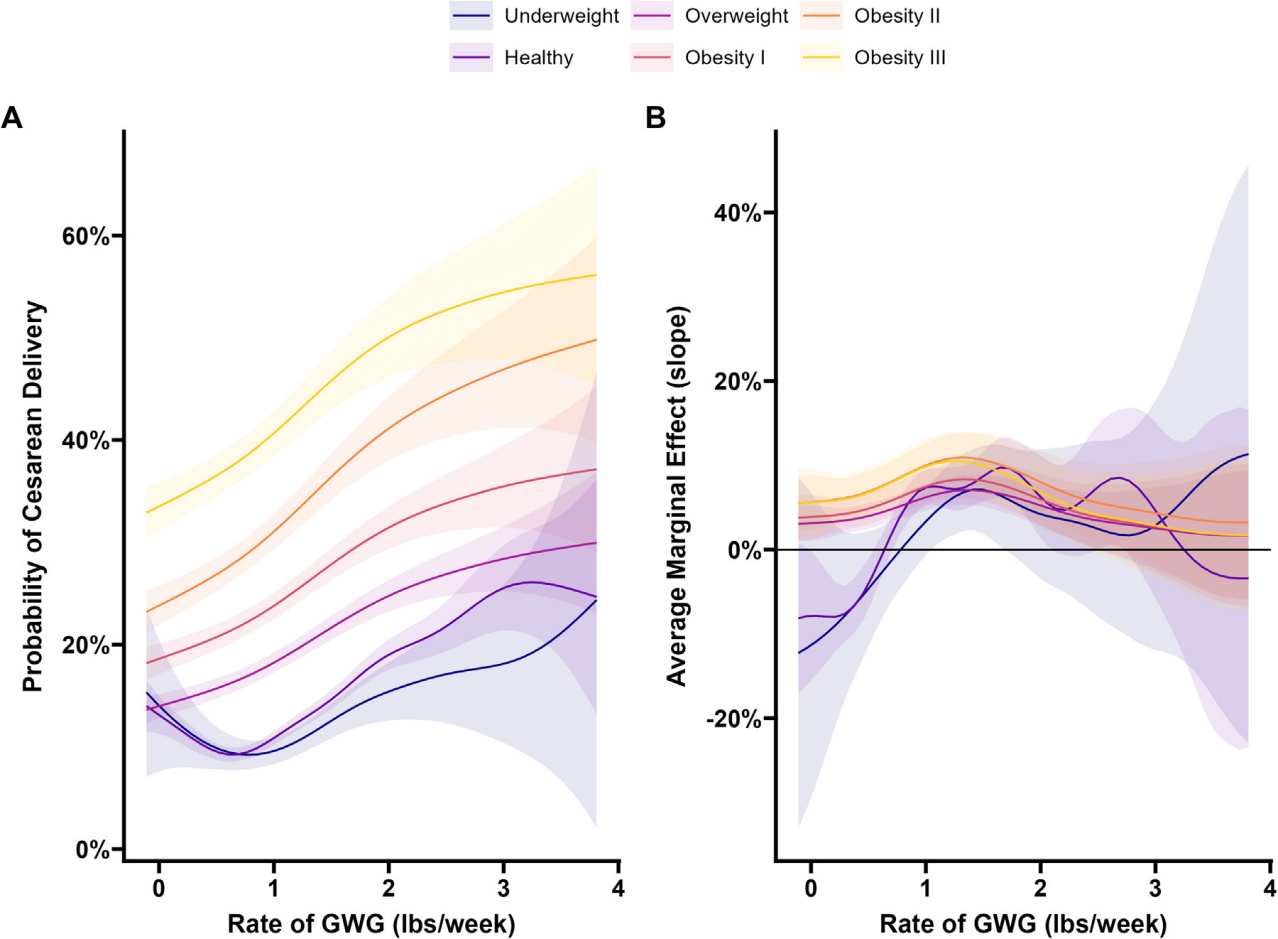
Visualization of the generalized additive model with gestational weight gain **A**, The predicted population average probability of cesarean delivery across the range of GWG rate. **B**, The partial derivatives of the slopes from the model of GWG rate. The error bands around each line represent 95% confidence intervals. *GWG*, gestational weight gain.

When assessing the first derivative of the spline slopes, the risk of cesarean delivery among women with a healthy prepregnancy BMI decreased as the rate of GWG increased until the individual reached a GWG of 0.7 lb/wk (Figure 2, B), a detail not captured when rate of GWG is categorized. Although the interaction term was nonsignificant, underweight mothers followed a pattern similar to that of healthy-weight mothers, but with a high degree of uncertainty in the underweight interaction smooth term (as indicated by the wide confidence intervals for the underweight group in Figure 2). However, among women with a prepregnancy BMI in the overweight or obese range, an increase in the GWG rate was associated with a higher risk of cesarean delivery (Figure 2).

McElfish. Gestational weight gain and increased risk of cesarean delivery. Am J Obstet Gynecol Glob Rep 2025.

## Comment

### Principal findings

Regardless of prepregnancy BMI, women with EGWG had a higher risk of cesarean delivery, which was confirmed in our sensitivity analysis of total GWG. Among women with a healthy prepregnancy BMI, the risk of cesarean delivery decreased as GWG increased with appropriate weight gain (ie, approximately 0.7 lb/wk of GWG), suggesting a potential protective effect of moderate weight gain for individuals with a healthy prepregnancy BMI. Although not significant, a similar trend was observed for individuals with underweight prepregnancy BMI. However, exceeding the appropriate weight gain was associated with increased risk of cesarean delivery. For women whose BMI was categorized as overweight or obese, any increase in the GWG rate was linked to a higher cesarean delivery risk. Furthermore, we found that among women with prepregnancy BMI in the overweight or obese categories, any GWG increased the risk of cesarean delivery, suggesting the need to further evaluate the clinical implications of the current GWG recommendations among these populations. Our analysis showed that the risk of cesarean delivery increased with higher prepregnancy BMI in this large, multiethnic sample.^35^, ^36^, ^37^

### Results in the context of what is known

Although the overall finding that EGWG was associated with cesarean delivery is consistent with prior literature,^17^, ^18^, ^19^^,^^38^^,^^39^ our analysis provides a comprehensive list of prepregnancy BMI categories (not only overweight and obesity). This analysis also examined cesarean delivery outcomes using rate of GWG categorizations and treating the rate of GWG as a continuous variable.

### Clinical implications

This study has several implications for clinical practice. Our analysis focused on the risk associated with GWG rate in the second and third trimesters, and there is an opportunity for this risk to be modified with interventions that begin alongside or are delivered as part of routine prenatal care. However, several studies have found that a low percentage of obstetrical providers counsel their patients using NAM guidelines for GWG,^40^, ^41^, ^42^, ^43^, ^44^, ^45^, ^46^ and that the revised 2009 guidelines (which lowered GWG recommendations for higher BMI categories) did not reduce EGWG for women in higher BMI categories.^47^ The Perinatal Quality Collaboratives and Alliance for Innovation on Maternal Health (AIM) bundle to reduce cesarean deliveries does not explicitly include GWG counseling or efforts to reduce EGWG. AIM bundles are also powerful quality improvement tools, and our findings suggest that GWG counseling should be added to AIM bundles.

### Research implications

Although studies have documented that providers often do not provide GWG counseling consistent with guidelines, there is a lack of research on why this occurs. Furthermore, we found no published literature on provider-level interventions for increasing guideline-consistent GWG recommendations. Interventions to reduce EGWG, including those focused on diet, physical activity, or a combination of diet and physical activity, have demonstrated effectiveness.^48^^,^^49^ However, these interventions primarily included women least affected by EGWG (affluent, urban, and suburban White women), leaving a significant gap in the research literature on effective interventions to reduce EGWG among women most at risk.^49^, ^50^, ^51^, ^52^ Future research is needed to test provider-level interventions (such as AIM bundles) to increase GWG counseling consistent with NAM guidelines, and further research is needed to examine effective interventions to reduce EGWG among diverse, low-income, and rural women.

### Strengths and limitations

This study has some limitations. The analysis used data from birth records to calculate GWG and prepregnancy BMI.^53^ Some research has shown maternal weight data derived from birth certificates to have poor agreement with medical record data.^54^^,^^55^ Errors in birth record–derived prepregnancy weight could lead to misclassification of GWG, particularly for women who have late or no prenatal care and for women with very low and very high GWG. Given that women are more likely to underestimate their prepregnancy weight,^56^ the total weight gain and rate of weight gain in our study may be overstated. The analysis examined the association between GWG and cesarean delivery, but these associations do not fully establish causality. There may be unmeasured confounders that are not available in the birth record data (eg, socioeconomic status and provider practice norms). As stated in the ACOG guidelines, the associations between maternal obesity class, GWG, and maternal and newborn outcomes are complex.^57^ We made efforts to improve the strength of our findings by focusing on a low-risk population, including potential covariates, using 2 separate measures of GWG, and using 2 separate analytical approaches (categorical and continuous) to assess the association. Our results were also strengthened because we used the rate of GWG categorizations and examined the rate of GWG as a continuous variable; these results were confirmed by sensitivity analysis using total GWG.

Although there are limitations to the analysis, the results provide valuable insights into the negative effects of EGWG on mothers regardless of prepregnancy BMI category.

### Conclusion

The United States has some of the poorest maternal and infant outcomes of any high-income nation.^1^^,^^2^ The United States also has a higher proportion of cesarean delivery than other countries.^8^, ^9^, ^10^ Medically unnecessary cesarean delivery can have negative implications for both mothers and infants.^7^^,^^12^ Reducing cesarean delivery is a shared goal of the Centers for Disease Control and Prevention Healthy People 2030, ACOG, and the AIM.^13^^,^^14^ Although many of the nonclinical factors associated with cesarean delivery (maternal age, race, socioeconomic status, and insurance coverage) and clinical factors associated with cesarean delivery (prepregnancy BMI and prepregnancy health conditions) are difficult to modify, our findings suggest that weight gain during pregnancy may be a key modifiable risk factor to reduce the need for cesarean delivery, ultimately improving maternal and infant health outcomes. Given our findings of increased cesarean deliveries associated with EGWG across BMI categories, addressing EGWG is likely to have significant implications across populations.

## CRediT authorship contribution statement

**Pearl A. McElfish:** Writing – original draft, Funding acquisition, Conceptualization. **Britni L. Ayers:** Writing – review & editing, Writing – original draft. **Nicola L. Hawley:** Writing – review & editing, Writing – original draft, Conceptualization. **Aaron Caldwell:** Writing – review & editing, Writing – original draft, Formal analysis, Data curation. **Austin Porter:** Writing – review & editing, Writing – original draft, Conceptualization. **Michael D. Macechko:** Writing – review & editing. **Donya Watson:** Writing – review & editing. **Jennifer A. Callaghan-Koru:** Writing – review & editing. **James P. Selig:** Writing – review & editing, Formal analysis. **Jennifer A. Andersen:** Writing – review & editing. **Nirvana Manning:** Writing – review & editing. **Lanita White:** Writing – review & editing. **Enrique Gomez-Pomar:** Writing – review & editing, Conceptualization. **Clare C. Brown:** Writing – review & editing, Writing – original draft, Conceptualization.

## Appendix Supplementary materials

Image, application 1

## Footnotes

Supplementary material associated with this article can be found in the online version at doi:10.1016/j.xagr.2025.100445.
