# Supplementary material for: Gestational weight gain and increased risk of cesarean delivery across body mass index categories
Source: AJOG Glob Rep. 2025 Jan 17;5(1):100445. doi: 10.1016/j.xagr.2025.100445 (PMC11869019; doi:10.1016/j.xagr.2025.100445)
Supplement: Supplementary file 1 [file mmc1.pdf]

Appendix: Cesarean Risk and EGWG in Arkansas

Table of contents

Introduction 2

Definitions of GWG status 3

- Rate of GWG 3
- Total GWG 3

Rate of GWG 4

- Comparisons 5
  - Main Effect of Weight Gain Type 5
  - Main Effect of BMI 6
- Model Parameters 7

Total GWG 9

- Comparisons 10
  - Main Effect of Weight Gain Type 10
  - Main Effect of BMI 11
- Model Parameters 12

Continuous Modeling of Gestational Weight Gain 14

- Rate of GWG - LOESS 14
- Rate of GWG 14

Introduction

Data was restricted to singleton mothers in Arkansas with a birth order (parity) of 1, with a gestational age at birth of 37 weeks or greater, not presenting as breech at birth (as close as we can get to nulliparous, term, singleton, vertex).

Risk Factors

The following factors were included in the model as covariates.

- Diabetes (pre and gestational) - `pre_diab` & `gest_diab`
- Hypertension (pre and gestational) - `pre_hype` & `gest_hype`
- Induction of Labor - `ld_indl`
- Mother's Age - `mother_age`
- Mother's Race - `mrace_main`
- Year of Birth - `year`
- Gestational Age - `gest_age`
- BMI (interaction with weight gain category) - `bmi_category`
- Metro/Nonmetro County - `metro`
- Payer - `payment`

Definitions of GWG status

Rate of GWG

Rate of GWG was calculated as the following

$$RateGWG = \frac{TotalGWG - 2.75lbs}{GA - 12weeks}$$

Healthy Range:

- Underweight: 1-1.3 lbs/week
- Normal: 0.8 - 1.0 lbs/week
- Overweight: 0.5 - 0.7 lbs/week
- Obese: 0.4 - 0.6 lbs/week

Total GWG

Additionally, as a robustness check, the GWG was defined by total GWG; results are consistent between both methods.

Healthy Range:

- Underweight: 28-40 lbs
- Normal: 25-35 lbs
- Overweight: 15-25 lbs
- Obese: 11-20 lbs

Rate of GWG

Below is the population average *predicted* probability, from the logistic regression model, of c-section with GWG defined by rate of GWG.

acog_wg_rate	bmi_cat	estimate	std.error
Excessive	Normal	19.75%	0.268%
Excessive	Overweight	28.53%	0.367%
Insufficient	Obesity I	27.03%	0.904%
Excessive	Obesity I	35.62%	0.516%
Insufficient	Normal	13.60%	0.356%
Healthy	Normal	14.63%	0.464%
Insufficient	Underweight	12.31%	0.964%
Healthy	Obesity I	31.45%	1.315%
Insufficient	Obesity II	34.01%	1.123%
Excessive	Obesity III	56.84%	0.976%
Healthy	Underweight	13.17%	1.109%
Healthy	Overweight	23.50%	0.929%
Excessive	Obesity II	45.36%	0.796%
Healthy	Obesity III	52.21%	2.060%
Insufficient	Overweight	20.46%	0.704%
Insufficient	Obesity III	48.48%	1.171%
Excessive	Underweight	17.93%	1.051%
Healthy	Obesity II	41.29%	1.850%

Comparisons

The comparisons listed below were made using the estimated marginal means from the logistic regression model (*marginaleffects* R package). These are the population average effects (averaged over all participants).

Main Effect of Weight Gain Type

Table 2: Absolute Risk Difference (%) between GWG

contrast	estimate	std.error	statistic	p.value	conf.low	conf.high
mean(Excessive) - mean(Healthy)	0.048	0.004	10.679	<0.001	0.039	0.057
mean(Excessive) - mean(Insufficient)	0.071	0.004	20.142	<0.001	0.064	0.078
mean(Healthy) - mean(Insufficient)	0.023	0.005	4.616	<0.001	0.013	0.033

Main Effect of BMI

Table 3: Absolute Risk Difference (%) between BMI categories

contrast	estimate	std.error	statistic	p.value	conf.low	conf.high
mean(Healthy) - mean(Underweight)	0.006	0.008	0.754	0.451	-0.010	0.022
mean(Obesity I) - mean(Overweight)	0.060	0.005	11.435	<0.001	0.050	0.070
mean(Obesity II) - mean(Obesity I)	0.075	0.007	10.113	<0.001	0.061	0.090
mean(Obesity III) - mean(Obesity II)	0.096	0.010	9.942	<0.001	0.077	0.115
mean(Overweight) - mean(Healthy)	0.073	0.004	19.584	<0.001	0.066	0.081

Model Parameters

	Estimate	Standard Error	z value	Pr(>|z|)	
(Intercept)	-1.805	0.053	-34.171	0.0000	***
acog_wg_rate.L	0.305	0.082	3.715	0.0002	***
acog_wg_rate.Q	0.116	0.093	1.247	0.2125	
bmi_categoryHealthy	0.039	0.054	0.726	0.4679	
bmi_categoryOverweight	0.503	0.056	8.941	0.0000	***
bmi_categoryObesity I	0.823	0.058	14.225	0.0000	***
bmi_categoryObesity II	1.163	0.061	19.034	0.0000	***
bmi_categoryObesity III	1.579	0.063	25.225	0.0000	***
year.L	-0.008	0.025	-0.301	0.7632	
year.Q	-0.108	0.025	-4.378	0.0000	***
year.C	0.072	0.025	2.864	0.0042	**
year ^4	0.040	0.025	1.608	0.1078	
year ^5	-0.019	0.025	-0.761	0.4467	
year ^6	-0.080	0.025	-3.176	0.0015	**
year ^7	-0.047	0.025	-1.835	0.0665	.
year ^8	-0.008	0.025	-0.326	0.7447	
mrace_mainHispanic	-0.010	0.031	-0.323	0.7470	
mrace_mainBlack	0.207	0.023	9.012	0.0000	***
mrace_mainAsian	0.213	0.052	4.088	0.0000	***
mrace_mainAIAN	0.170	0.105	1.617	0.1059	
mrace_mainNHPI	0.444	0.108	4.124	0.0000	***
mrace_mainMultiracial	0.152	0.059	2.569	0.0102	*

	Estimate	Standard Error	z value	Pr(>|z|)	
paymentOther	-0.125	0.074	-1.698	0.0894	.
paymentPrivate Insurance	-0.154	0.019	-7.930	0.0000	***
paymentSelf-Pay	-0.270	0.055	-4.922	0.0000	***
metro.L	-0.077	0.013	-6.099	0.0000	***
I(gest_age - 40)	0.027	0.005	5.105	0.0000	***
pre_diab	0.983	0.101	9.722	0.0000	***
pre_hype	0.438	0.060	7.310	0.0000	***
gest_diab	0.312	0.039	8.077	0.0000	***
gest_hype	0.376	0.028	13.254	0.0000	***
ld_indl	0.306	0.017	17.572	0.0000	***
I(mother_age - 24)	0.052	0.002	28.907	0.0000	***
acog_wg_rate.L:bmi_categoryHealthy	0.007	0.086	0.086	0.9317	
acog_wg_rate.Q:bmi_categoryHealthy	0.002	0.099	0.016	0.9869	
acog_wg_rate.L:bmi_categoryOverweight	-0.012	0.089	-0.139	0.8891	
acog_wg_rate.Q:bmi_categoryOverweight	-0.062	0.105	-0.594	0.5528	
acog_wg_rate.L:bmi_categoryObesity I	-0.025	0.090	-0.282	0.7779	
acog_wg_rate.Q:bmi_categoryObesity I	-0.120	0.109	-1.108	0.2681	
acog_wg_rate.L:bmi_categoryObesity II	0.023	0.093	0.242	0.8086	
acog_wg_rate.Q:bmi_categoryObesity II	-0.176	0.116	-1.517	0.1293	
acog_wg_rate.L:bmi_categoryObesity III	-0.078	0.094	-0.834	0.4045	
acog_wg_rate.Q:bmi_categoryObesity III	-0.098	0.119	-0.824	0.4097	

*Signif. codes: 0 <= '***' < 0.001 < '**' < 0.01 < '*' < 0.05*

(Dispersion parameter for binomial family taken to be 1)

Null deviance: 9.447e+04 on 82801 degrees of freedom

Residual deviance: 8.768e+04 on 82759 degrees of freedom

Total GWG

Below is the population average *predicted* probability, from the logistic regression model, of c-section with GWG defined by *total* GWG (total weight gained during pregnancy). The results closely match those of the rate of GWG.

acog_wg_weight	bmi_cat	estimate	std.error
Excessive	Normal	21.445%	0.3323%
Excessive	Overweight	29.206%	0.3922%
Insufficient	Obesity I	27.100%	1.0194%
Excessive	Obesity I	35.962%	0.5337%
Healthy	Normal	15.000%	0.3084%
Insufficient	Normal	14.016%	0.3764%
Insufficient	Underweight	12.957%	1.1101%
Healthy	Obesity I	30.158%	0.9591%
Insufficient	Obesity II	34.321%	1.2542%
Healthy	Obesity III	51.934%	1.4857%
Healthy	Underweight	13.002%	0.9036%
Healthy	Overweight	23.064%	0.6393%
Excessive	Obesity II	45.880%	0.8293%
Excessive	Obesity III	57.650%	1.0211%
Healthy	Obesity II	38.609%	1.3230%
Insufficient	Overweight	20.381%	0.7695%
Insufficient	Obesity III	47.379%	1.2841%
Excessive	Underweight	18.314%	1.1550%

Comparisons

The comparisons listed below were made using the estimated average means from the logistic regression model.

Main Effect of Weight Gain Type

Table 6: Absolute Risk Difference (%) between GWG

contrast	estimate	std.error	statistic	p.value	conf.low	conf.high
mean(Excessive) - mean(Healthy)	0.0608	0.0036	17.0578	<0.001	0.0538	0.0678
mean(Excessive) - mean(Insufficient)	0.0785	0.0039	20.1495	<0.001	0.0708	0.0861
mean(Healthy) - mean(Insufficient)	0.0177	0.0043	4.1164	<0.001	0.0093	0.0261

Main Effect of BMI

Table 7: Absolute Risk Difference (%) between BMI categories

contrast	estimate	std.error	statistic	p.value	conf.low	conf.high
mean(Healthy) - mean(Underweight)	0.0136	0.0078	1.7478	0.081	-0.0017	0.0289
mean(Obesity I) - mean(Overweight)	0.0576	0.0053	10.8158	<0.001	0.0471	0.0680
mean(Obesity II) - mean(Obesity I)	0.0740	0.0075	9.8786	<0.001	0.0593	0.0886
mean(Obesity III) - mean(Obesity II)	0.0996	0.0096	10.3882	<0.001	0.0808	0.1184
mean(Overweight) - mean(Healthy)	0.0658	0.0038	17.3051	<0.001	0.0583	0.0732

Model Parameters

	Estimate	Standard Error	z value	Pr(>|z|)	
(Intercept)	-1.785	0.052	-34.097	0.0000	***
acog_wg_weight.L	0.274	0.090	3.048	0.0023	**
acog_wg_weight.Q	0.169	0.084	2.018	0.0436	*
bmi_categoryHealthy	0.072	0.053	1.370	0.1707	
bmi_categoryOverweight	0.485	0.055	8.853	0.0000	***
bmi_categoryObesity I	0.787	0.056	13.976	0.0000	***
bmi_categoryObesity II	1.115	0.059	19.014	0.0000	***
bmi_categoryObesity III	1.548	0.060	25.966	0.0000	***
year.L	-0.011	0.025	-0.417	0.6770	
year.Q	-0.106	0.025	-4.300	0.0000	***
year.C	0.073	0.025	2.921	0.0035	**
year ^4	0.043	0.025	1.731	0.0835	.
year ^5	-0.021	0.025	-0.828	0.4076	
year ^6	-0.076	0.025	-3.011	0.0026	**
year ^7	-0.048	0.025	-1.874	0.0610	.
year ^8	-0.006	0.025	-0.258	0.7964	
mrace_mainHispanic	-0.006	0.031	-0.211	0.8327	
mrace_mainBlack	0.209	0.023	9.083	0.0000	***
mrace_mainAsian	0.219	0.052	4.192	0.0000	***
mrace_mainAIAN	0.169	0.105	1.613	0.1067	
mrace_mainNHPI	0.437	0.108	4.065	0.0000	***
mrace_mainMultiracial	0.146	0.059	2.469	0.0136	*

	Estimate	Standard Error	z value	Pr(>|z|)	
paymentOther	-0.121	0.074	-1.648	0.0994	.
paymentPrivate Insurance	-0.149	0.019	-7.640	0.0000	***
paymentSelf-Pay	-0.268	0.055	-4.889	0.0000	***
metro.L	-0.076	0.013	-6.049	0.0000	***
I(gest_age - 40)	0.019	0.005	3.622	0.0003	***
pre_diab	0.978	0.101	9.678	0.0000	***
pre_hype	0.433	0.060	7.225	0.0000	***
gest_diab	0.315	0.039	8.157	0.0000	***
gest_hype	0.366	0.028	12.886	0.0000	***
ld_indl	0.300	0.017	17.186	0.0000	***
I(mother_age - 24)	0.053	0.002	29.150	0.0000	***
acog_wg_weight.L:bmi_categoryHealthy	0.079	0.094	0.848	0.3964	
acog_wg_weight.Q:bmi_categoryHealthy	-0.017	0.088	-0.190	0.8497	
acog_wg_weight.L:bmi_categoryOverweight	0.040	0.097	0.407	0.6839	
acog_wg_weight.Q:bmi_categoryOverweight	-0.091	0.092	-0.995	0.3197	
acog_wg_weight.L:bmi_categoryObesity I	0.010	0.099	0.101	0.9193	
acog_wg_weight.Q:bmi_categoryObesity I	-0.115	0.095	-1.201	0.2296	
acog_wg_weight.L:bmi_categoryObesity II	0.054	0.102	0.532	0.5949	
acog_wg_weight.Q:bmi_categoryObesity II	-0.123	0.100	-1.225	0.2205	
acog_wg_weight.L:bmi_categoryObesity III	0.015	0.102	0.150	0.8805	
acog_wg_weight.Q:bmi_categoryObesity III	-0.150	0.102	-1.467	0.1424	

*Signif. codes: 0 <= ’***’ < 0.001 < ’**’ < 0.01 < ’*’ < 0.05*

(Dispersion parameter for binomial family taken to be 1)

Null deviance: 9.447e+04 on 82801 degrees of freedom

Residual deviance: 8.758e+04 on 82759 degrees of freedom

Continuous Modeling of Gestational Weight Gain

Using a general additive mode with thin-plate splines, we can look at the *continuous* effect of GWG on c-section risk.

Rate of GWG - LOESS

Local polynomial regression fitting can also be used to plot the raw averages (probabilities) in the data. This produces similar curves to the model but unadjusted for other factors.

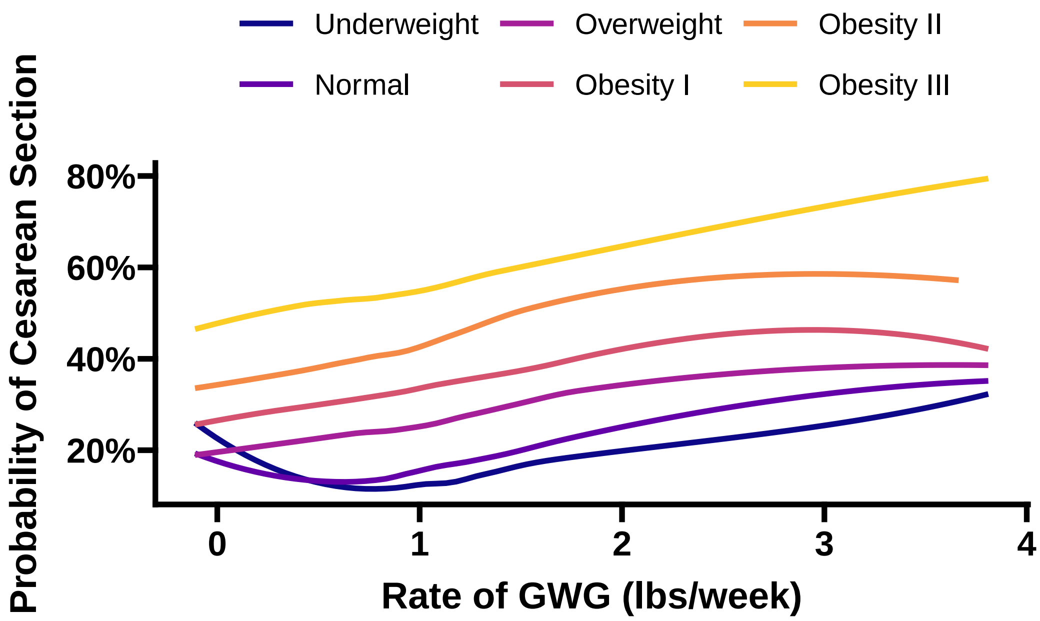

Rate of GWG

Component	Term	Estimate	Std Error	t-value	p-value
	(Intercept)	-1.786	0.059	-30.480	0.0000 ***
	bmi_categoryHealthy	0.106	0.059	1.807	0.0707 .
	bmi_categoryOverweight	0.601	0.059	10.155	0.0000 ***
	bmi_categoryObesity I	0.937	0.060	15.533	0.0000 ***
	bmi_categoryObesity II	1.307	0.064	20.533	0.0000 ***

Component	Term	Estimate	Std Error	t-value	p-value	
	bmi_categoryObesity III	1.723	0.067	25.632	0.0000	***
	year.L	0.000	0.025	0.006	0.9952	
	year.Q	-0.108	0.025	-4.360	0.0000	***
	year.C	0.073	0.025	2.913	0.0036	**
	year ^4	0.041	0.025	1.655	0.0980	.
	year ^5	-0.017	0.025	-0.662	0.5077	
	year ^6	-0.073	0.025	-2.910	0.0036	**
	year ^7	-0.049	0.026	-1.913	0.0558	.
	year ^8	-0.010	0.025	-0.387	0.6986	
	mrace_mainHispanic	0.010	0.031	0.337	0.7358	
	mrace_mainBlack	0.212	0.023	9.215	0.0000	***
	mrace_mainAsian	0.244	0.052	4.674	0.0000	***
	mrace_mainAIAN	0.157	0.105	1.488	0.1366	
	mrace_mainNHPI	0.442	0.108	4.106	0.0000	***
	mrace_mainMultiracial	0.126	0.059	2.126	0.0335	*
	paymentOther	-0.115	0.074	-1.557	0.1195	
	paymentPrivate Insurance	-0.132	0.019	-6.765	0.0000	***
	paymentSelf-Pay	-0.265	0.055	-4.828	0.0000	***
	metro.L	-0.074	0.013	-5.909	0.0000	***
	I(gest_age - 40)	0.035	0.005	6.614	0.0000	***
	pre_diab	0.967	0.101	9.538	0.0000	***
	pre_hype	0.420	0.060	6.990	0.0000	***
	gest_diab	0.312	0.039	8.065	0.0000	***

Component	Term	Estimate	Std Error	t-value	p-value	
	gest_hype	0.341	0.029	11.969	0.0000	***
	ld_indl	0.293	0.017	16.744	0.0000	***
	I(mother_age - 24)	0.053	0.002	29.384	0.0000	***
**Component**	**Term**	**edf**	**Ref. df**	**F-value**	**p-value**	
B. smooth terms	s(rate_gwg)	3.665	4.560	56.677	0.0000	***
	s(rate_gwg):bmi_categoryUnderweight	3.572	4.497	7.637	0.1372	
	s(rate_gwg):bmi_categoryHealthy	6.090	7.190	61.863	0.0000	***
	s(rate_gwg):bmi_categoryOverweight	1.000	1.001	0.027	0.8699	
	s(rate_gwg):bmi_categoryObesity I	1.000	1.001	0.006	0.9415	
	s(rate_gwg):bmi_categoryObesity II	1.000	1.001	0.839	0.3600	
	s(rate_gwg):bmi_categoryObesity III	0.001	0.001	0.000	0.9942	

Signif. codes: 0 <= '***' < 0.001 < '**' < 0.01 < '*' < 0.05

Adjusted R-squared: 0.0895, Deviance explained 0.0766

fREML : 117555.548, Scale est: 1.000, N: 82802
